# Primary Brainstem Lymphoma: A Population-Based Study

**DOI:** 10.3389/fsurg.2022.829048

**Published:** 2022-07-06

**Authors:** Junyu Chen, Bo Cen, Fei Hu, Yong Qiu, Guomin Xiao, Junge Zhou, Xiujian Ma, Fangcheng Zhang

**Affiliations:** ^1^ General Hospital of the Yangtze River Shipping, Wuhan Brain Hospital, Wuhan, China; ^2^ German Cancer Research Center (DKFZ), DKFZ-ZMBH Alliance, Heidelberg, Germany; ^3^ Wuhan Union Hospital, Tongji Medical College, Huazhong University of Science and Technology, Wuhan, China

**Keywords:** primary brainstem lymphoma, survival, SEER, surgery, chemotherapy

## Abstract

**Background:**

Primary brainstem lymphoma (PBSL) is rare and malignant. An understanding of this disease is lacking. We aimed to characterize clinical features, estimate survival, and explore survival-related factors of PBSL.

**Methods:**

Patients with a histological diagnosis of primary lymphoma in the brainstem (C71.7) from 1975 to 2016 were retrieved from the Surveillance, Epidemiology, and End Results (SEER) program. Log-rank tests and univariate and multivariate Cox proportional hazard analyses were used to identify survival-related factors.

**Results:**

PBSL constituted 2.7% of brainstem malignancies. The median age of the PBSL patients was 59.5 years. Diffuse large B cell lymphoma (*n* = 49, 84.5%) was the most prevalent histology among the 58 cases with reported specific lymphoma subtype. The majority of PBSLs were localized (*n* = 46, 52.3%), at low Ann Arbor Stage (I/II, *n* = 63, 70.5%), and presented as a single primary (*n* = 71, 80.7%). Chemotherapy was applied in 50 (56.8%) cases. Three-year overall survival (OS) and disease-specific survival (DSS) rates were 42.7% and 53.5%, respectively. Multivariate analyses showed that independent predictive/prognostic factors for OS were age (*P* = 0.004), tumor number (*P* = 0.029), and chemotherapy (*P* = 0.001); DSS-related factors only included age (*P* = 0.014) and chemotherapy (*P* = 0.008).

**Conclusions:**

We estimated survival rates for PBSL patients. Factors associated with OS and DSS were also identified. Our findings addressed the importance of chemotherapy in treating PBSL patients.

## Importance of the Study

Our study represented the first study to comprehensively investigate PBSL based on a population dataset. We identified several independent predictors for overall survival (OS) and disease-specific survival (DSS). Demographical features, tumor-related characteristics, and therapeutical details were reported.

## Introduction

Primary central nervous system lymphoma (PCNSL) is an uncommon variant of extranodal non-Hodgkin lymphoma (NHL). This disease involves the eye, brain, spinal cord, etc. but without evidence of systemic disease. It accounts for 1.9% of all primary central nervous system (CNS) neoplasms and 6.4% of primary CNS malignancies (1). Patients with acquired or congenital immunodeficient disease are susceptible to PCNSL (2). The current treatment strategy consisted of biopsy followed by high-dose methotrexate (HDMTX)-based chemotherapy (3). The efficacy of craniotomy is controversial (3, 4). However, the outcomes of these patients remain dismal, with 5-year overall survival being 37.6% (1).

Primary brainstem lymphoma (PBSL) was rarely encountered in clinical practice. However, PCNSL located at the brainstem predicts more severe surgical complications (5). At present, there are only several case reports from a single institute (6–18). The existing published studies failed to estimate the survival of PBSL, let alone explore survival-related factors. As nationwide datasets, the Surveillance, Epidemiology, and End Results (SEER) datasets are an ideal platform to study rare malignant entities, e.g., PBSL (19). Previous studies leveraged the SEER dataset to characterize primary spinal lymphoma (20). However, no research yet applies the SEER dataset to study PBSL. Clinical characteristics and prognostic/predictive factors of PBSL patients remain poorly understood. We aim to elucidate survival and survival-associated factors for overall survival (OS) and disease-specific survival (DSS) based on a population-based dataset.

## Methods

### Case Selection From the SEER Database

**Figure 1:**
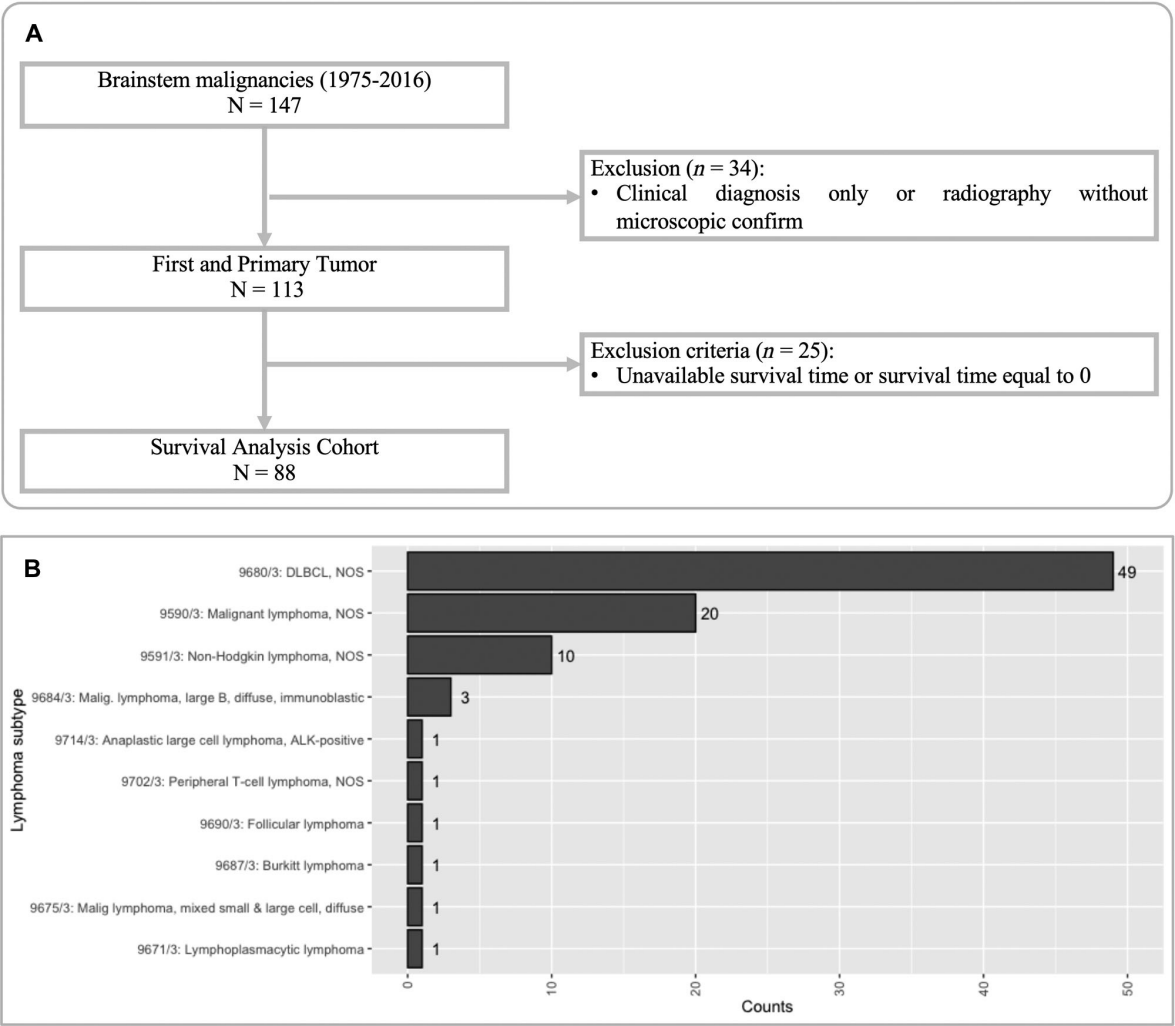
(**A**) Flow diagram of study cohort selection. (**B**) Histological variants of primary brainstem lymphoma (PBSL). ALK, anaplastic lymphoma kinase; DLBCL, diffuse large B cell lymphoma; NOS, not otherwise specified.

The SEER program of the National Cancer Institute is an authoritative source for cancer statistics in the United States. Because all the information is collected in a de-identified manner, the Institutional Review Board's approval was exempted. This study extracted data from incidence-SEER 18 Registries Custom Data (with additional treatment fields), Nov 2018 Sub (1975–2016 varying). This dataset covers 27.8% of the US population (based on the 2010 census). To obtain quantified PBSL cases, we queried the program by defining the first ICD-O-3 primary site (brainstem, C71.7) and then the histology record (lymphoma). Our selection strategy is detailed in Figure 1, where the goal is to obtain histologically confirmed PBSL cases and exclude patients without qualified survival time. SEER*Stat (Version 8.3.9, https://seer.cancer.gov/seerstat/) was used to retrieve the data.

### Definitions of Variables

OS and DSS were our main and additional endpoints, respectively. Although both marked the length of time from the date of diagnosis to the date of death, OS took into account deaths irrespective of causes; in contrast, when measuring DSS, patients who died from causes other than PBSL were uncounted. We divided surgery extent into gross total resection (GTR), partial resection (PR), and no surgery, following the decoding strategy described by Dudley et al. (21) The Arbor staging system is widely applied to stage lymphoma anatomically (22) and is reclassified into low stage (stage I/II) and high stage (stage III/IV). The sequence number informs the sequence of PBSL among all neoplasms over the patient's lifetime. We replaced the sequence number in SEER with tumor number, where “one primary only” in the sequence number was defined as a single lesion in tumor number and others as multiple lesions.

Meanwhile, we dichotomized diagnosis year (1975–1996 vs. 1997–2016), sex (female vs. male), marital status (married vs. unmarried/unknown), lymphoma type (diffuse B cells vs. others), stage (localized vs. regional/distant), radiotherapy (with radiotherapy vs. no/unknown), and chemotherapy (with chemotherapy vs. no/unknown). Age at diagnosis (0–50, 51–70, and 71+) and race (white, black, and others) were classified into three groups.

### Statistical Analysis

All statistical analyses were conducted using R (version 3.8.0, 2019, http://www.r-project.org/). Since the distribution of age (*p* < 0.05) and follow-up time (*p* < 0.001) were non-normal in the *Shapiro–Wilk normality test*, a median with interquartile range (IQR) was used to describe the two parameters. Prognostic/predictive factors for both OS and DSS were evaluated by Cox proportional hazards regression. Kaplan–Meier curves were used to estimate survival and significant predictors of OS and DSS. Candidates with a *P*-value <0.05 in univariate Cox analysis were subject to multivariate analyses. All tests were two-sided, and a *P*-value <0.05 was considered statistically significant.

## Results

### Demographics and Clinical Characteristics of Cohort for Survival Analysis

We identified 143 PBSL cases, constituting 2.7% (*n* = 5,391) patients with brainstem malignancies. Finally, 88 PBSL cases met our criteria (Figure 1A). The median age of the cohort was 59.5 (IQR 45.8–68.3) years. A child (≤18 years) PBSL patient was found only in one case. The proportion of male patients was 52.3% (*n* = 46). The majority of the patients were white (*n* = 77, 87.5) and got married (*n* = 51, 58%).

All the 58 cases with reported specific lymphoma subtypes were NHL. B-cell-derived lymphoma (*n* = 55, 95.8%) represented the most dominant variants, most of which (*n* = 49, 84.5%) were diffuse large B cell lymphoma (DLBCL). There were two cases with T-cell lymphoma and one case with mixed small- and large-cell lymphoma. The details of the remaining variants are plotted in Figure 1B. Based on the Ann Arbor Staging system, stage I, stage II, and stage IV were, respectively, found in 62 (70.5%) cases, 1 (1.1%) case, and 13 (14.8%) cases, without PBSL at stage III being identified. Just over half the lesions (52.3%, *n* = 46) were localized at diagnosis. In terms of tumor number, single and multiple PBSLs were found in 71 (80.7%) and 17 (19.3%) patients (Table 1).

**Table 1:** Demographic, histological, and therapeutic details of primary brainstem lymphoma.

Variables	Overall (*N *= 88)	Variables	Overall (*N* = 88)
Diagnosis year		Ann_Arbor_Stage	
1997–2016	62 (70.5%)	Stage I	62 (70.5%)
1975–1996	26 (29.5%)	Stage II	1 (1.1%)
Age at diagnosis (years)		Stage IV	13 (14.8%)
Median	59.5	Missing	12 (13.6%)
Interquartile range	45.8–68.3	Tumor number	
Sex		Single	71 (80.7%)
Female	42 (47.7%)	Multiple	17 (19.3%)
Male	46 (52.3%)	Surgical extent	
Race		GTR	3 (3.4%)
White	77 (87.5%)	PR	27 (30.6%)
Black	5 (5.7%)	No surgery	51 (58.0%)
Other	6 (6.8%)	Missing	7 (8.0%)
Marital status		Radiotherapy	
Married	51 (58.0%)	Yes	49 (55.7%)
Single	23 (26.1%)	No/unknown	39 (44.3%)
Widowed	7 (8.0%)	Chemotherapy	
Divorced	2 (2.3%)	Yes	50 (56.8%)
Separated	1 (1.1%)	No/unknown	38 (43.2%)
Missing	4 (4.5%)		
Stage			
Localized	46 (52.3%)		
Reginal	1 (1.1%)		
Distant	10 (11.4%)		
Missing	31 (35.2%)		

### Therapeutical Details and Survival

One-third of patients (*n* = 30, 34.1%) with PBSL received surgical treatments, and GTR and PR were achieved in 3 (3.4%) and 27 (30.6%) cases, respectively. The diagnosis of PBSL was based on positive histology or exfoliative cytology. Radiotherapy and chemotherapy were applied in 49 (55.7%) and 50 (56.8%) cases, respectively.

**Figure 2:**
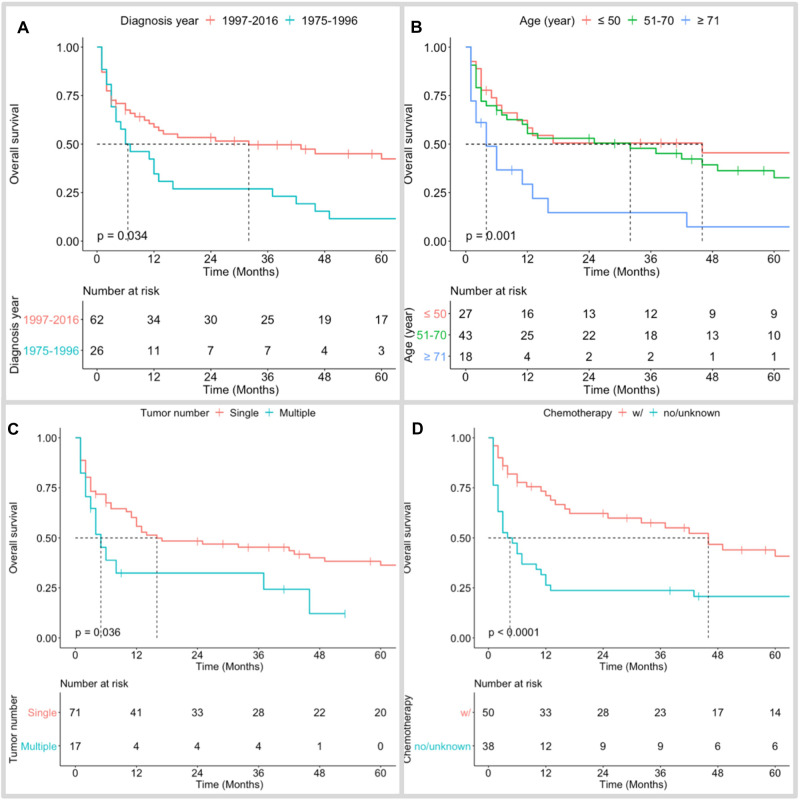
Kaplan–Meier overall survival curves of PBSL patients stratified by diagnosis year (**A**), age at diagnosis (**B**), tumor number (**C**), and use of chemotherapy (**D**).

The median follow-up time of the cohort was 12 (IQR 3–50) months. Only 20 (22.7%) patients survived. Among the 68 (77.3%) dead patients, 47 (53.4%) cases succumbed to PBSL, while the other 21 (23.9%) patients died from causes other than PBSL progression. The 1-, 3-, and 5-year OS rates of patients with PBSL were 51.3%, 42.7%, and 32.1%, respectively; the figures for DSS were 64.3%, 53.5%, and 45.0%, respectively (Figure 2).

### Prognostic/Predictive Factors

**Figure 3:**
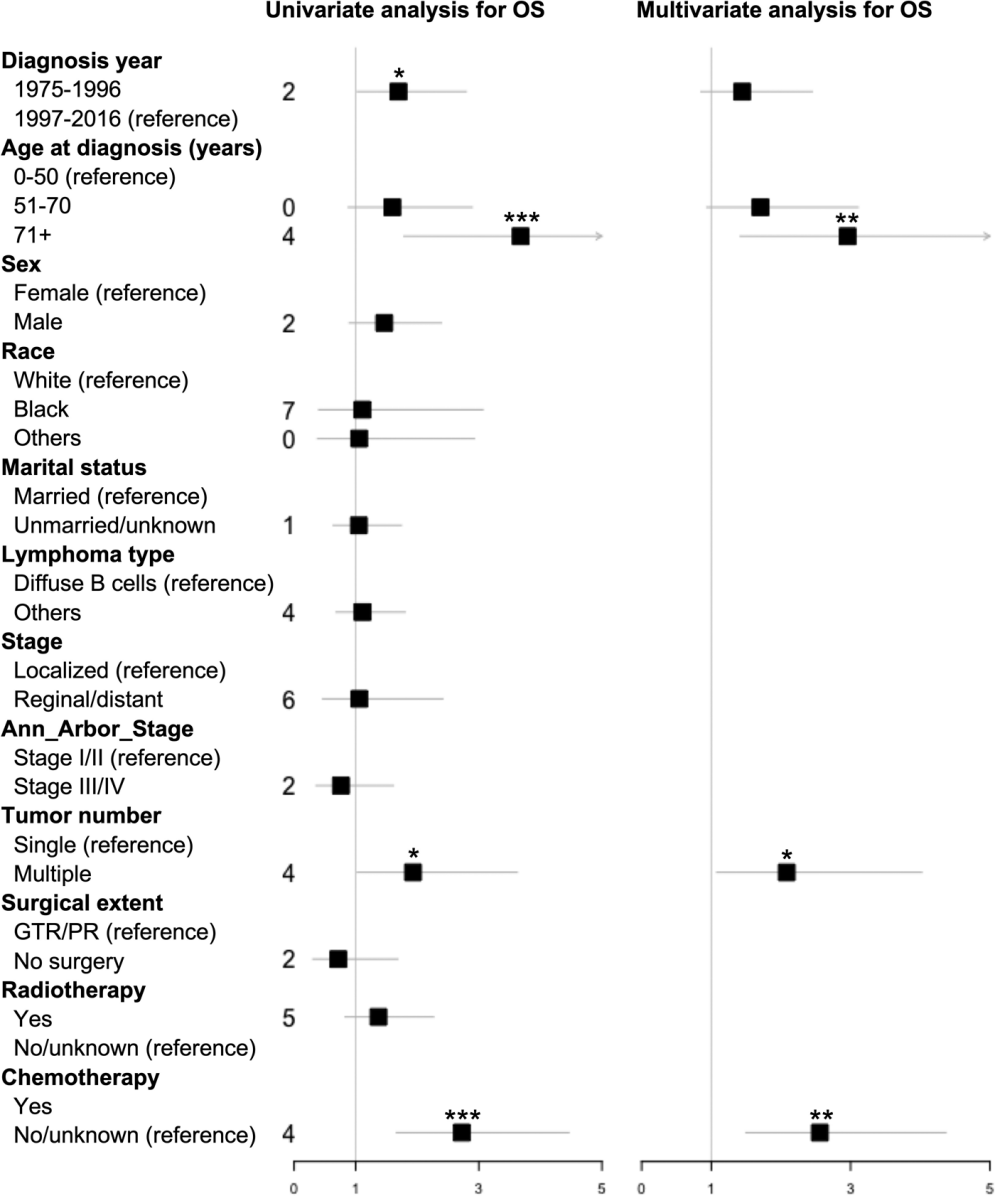
Forest plot based on univariate and multivariate Cox proportional hazard analysis of overall survival (OS) for PBSL patients. GTR, gross total resection; OS, overall survival; PR, partial resection. *, *P* < 0.05; **, *P* < 0.01; ***, *P* < 0.001.

Kaplan–Meier long-rank tests showed that patients diagnosed during 1975–1996 (Figure 2A), older age (Figure 2B), single PBSL (Figure 2C), and no use of chemotherapy (Figure 2D) were related to poor OS. In the univariate Cox analyses, consistent results were found. Subsequent multivariate tests identified several independent prognostic/predictive factors, including age (3-year OS of ≥71 years vs. ≤50 years = 14.6% vs. 50.6%, HR [95% CI] 2.957 [1.416, 6.178], *P* = 0.004), tumor number (3-year OS of single vs. multiple = 32.4% vs. 45.4%, HR [95% CI] 2.083 [1.078, 4.022], *P* = 0.029), and use of chemotherapy (3-year OS of no/unknown vs. with chemotherapy = 23.7% vs. 57.5%, HR [95% CI] 2.558 [1.498,4.368], *P* = 0.001, Figure 3).

In contrast, both DSS-associated factors, identified in Kaplan–Meier long-rank tests, namely, older age (Supplementary Figure 1A) and no use of chemotherapy (Supplementary Figure 1B), were verified in univariate and multivariate Cox analyses (Supplementary Figure 2). The 3-year DSS of ≥71 years vs ≤50 years (HR [95% CI] 2.938 [1.241,6.952], *P* = 0.014) was 22.6% vs. 60% and the figure for no/unknown vs. with chemotherapy (HR [95% CI] 2.288 [1.244,4.209], *P* = 0.008) was 35.9% vs. 64.5%.

## Discussion

To the best of our knowledge, our study represented the first study to investigate PBSL based on a population dataset comprehensively. With the power of the SEER database, we were able to collect 88 cases with PBSL to characterize the survival and prognostic/predictive factors of PBSL patients. In a nutshell, most patients were diagnosed in their sixth decade. Overall, only around half of PBSL patients survived 1 year after diagnosis. Age at diagnosis and sequence number were independent predictors for OS; age at diagnosis independently predicted DSS. The application of chemotherapy improved both OS and DSS.

### Clinical Features, Survival, and Prognostic Factors of PBSL

The common location of PCNS is the frontal lobes (23, 24). The proportion of PBSL in brainstem malignancies remains unknown. Our SEER data showed PBSL accounted for 2.7% of malignant brainstem tumors, indicating the rare nature of this entity. The literature search revealed only one study reported a child with a brainstem DLBCL (11). Likewise, our study unveiled that only one child suffered PBSL. PBSL mainly affected adults in their sixth decade with a slight male predominance. Parallel to previous case reports (6–18), our study confirmed that all PBSLs were NHLs. The prognosis of PBSL is poor. We found that major PBSL patients died during the study. Nearly half of patients died within 1 year after diagnosis; only 32.1% survived 3 years after diagnosis. This finding was similar to previous studies. Among the published 10 cases (6–9, 11–13, 16, 17) with survival status, 7 died within 1–18 months after diagnosis (7, 8, 11–13, 16, 17). In addition, we identified that older age was associated with poorer OS and DSS and multiple PBSLs with poorer OS.

### Predictive Factors of PBSL (Chemotherapy)

Combined modality therapy (chemotherapy plus radiotherapy) was an effective initial treatment for PCNSL (25). Accordingly, our SEER study findings showed that the use of chemotherapy prolonged both OS and DSS. However, chemotherapy regimens were unfeasible in the SEER database, precluding us from investigating the effectiveness of individual chemotherapy regimens. For these PBSLs reported previously, regimens contained HDMTX alone (11, 16), HDMTX plus cytarabine (AraR) (9), HDMTX plus HD dexamethasone (6), and HDMTX plus rituximab plus temozolomide (8).

### Radiotherapy in PBSL

Gamma knife surgery (GKS) was reported as a safe and effective approach for PCNSL (26). The accurately targeted ability of GKS made it an ideal method for managing brainstem lesions. Campbell et al. (7) treated PBSL patients by delivering GKS to tumor margin with a dose of 11 Gy. Two months later, they observed a 50% size reduction of the enhanced lesion. Kim et al. (11) also applied GKS at 12 Gy in addition to HDMTX and found the patient tolerated GKS well. Unfortunately, evaluation of the long-term benefit of radiotherapy treatment was inaccessible, as both cases died of causes other than PBSL in 6 and 10 months, respectively. Instead of GKS, Sato et al. (16) combined high-dose methotrexate and 30 Gy of whole-brain radiation and 10 Gy of focal radiation in 2 Gy fractions in two PBSL cases. One patient achieved remission 14.4 months into their study, and another patient succumbed to death 18 months after diagnosis. Due to the limited case number, we could not identify a significant role of radiotherapy. Therefore, the efficacy of radiotherapy needs to be investigated.

### Surgical Treatment for PBSL

According to recently released National Comprehensive Cancer Network (NCCN) guidelines, the treatment strategy for PCNSL patients incorporated biopsy with the least invasive approach, followed by a clinical trial or high-dose methotrexate-based regimen (27). Distinct from other CNS malignancies, cytoreductive surgery is not a standard of care for PCNSL (28). This resulted from no gain of survival benefit or even worsened prognoses after surgery (29, 30). However, Rae et al. recently reported that the survival of craniotomy-treated PCNSL patients was significantly improved compared to biopsy-treatment PCNSL patients in The National Cancer Database-Participant User File (NCDB) and SEER databases. They argued that previous studies were conducted in the pre-modern neurosurgical era when the assistance of intraoperative monitoring, neuro-navigation, and fluorescent-guided tumor resection was lacking (3). In their study, the authors further established a risk category (RC), where high RC involved deep location (e.g., brainstem and basal ganglia), age > 55 years, etc. Subsequently, they found that survival benefits could be obtained in low RC but not in high RC based on their institutional series (3). In specific to PBSL, our study failed to identify the benefits of OS or DSS from GTR/PR over no surgery. The brainstem is a critical anatomical structure, and injury involving the brainstem leads to disastrous consequences (5). Taken together, aggressive tumor resection of PBSL was not advocated.

### Study Limitations and Strengths

Our study contained several limitations. First, chemotherapy prolonged both OS and DSS, indicating its significant role in the survival of PBSL patients. However, the administration of specific compounds or combinations of compounds was unavailable in the SEER database. Second, it was known that patients with lymphoma subtypes had different outcomes. Due to its rarity, we could only identify nine cases with non- DLBCL. Merging these nine PBSLs into one subgroup (to achieve DLBCL vs. non-DLBCL) was not suitable and resulted in misleading as outcomes of these subtypes differed significantly (3). Thus, we did not investigate the impact of the lymphoma subtype on the survival of PBSL patients. Moreover, as a retrospective review, this study suffered from inherent selection bias. Nevertheless, the present study is the first to comprehensively characterize PBSL patients based on the largest cohort size. These findings are valued tremendously in terms of future clinical evaluation of survival and designation of clinical trials.

## Conclusions

Our study revealed the prognosis of PBSL was poor. Only around half of PBSL patients survived 1 year after diagnosis. While the sequence number affected OS but not DSS, age influenced both OS and DSS. The use of chemotherapy prolonged OS and DSS, addressing the significance of chemotherapy in treating PBSL patients. Future studies should focus on investigating effective chemotherapeutical regimes.

## Data Availability

The original contributions presented in the study are included in the article/Supplementary Material; further inquiries can be directed to the corresponding author/s.
