# Supplementary material for: Primary Brainstem Lymphoma: A Population-Based Study
Source: Front Surg. 2022 Jul 6;9:829048. doi: 10.3389/fsurg.2022.829048 (PMC9299248; doi:10.3389/fsurg.2022.829048)
Supplement: Supplementary file 1 [file Table_1_v1.docx]

| Table 1 Demographic, histological and therapeutic details of primary brainstem lymphoma | | | | |
| --- | --- | --- | --- | --- |
| **Variables** | Overall (N= 88) |  | **Variables** | Overall (N= 88) |
| **Diagnosis year** |  |  | **Ann_Arbor_Stage** |  |
| 1997-2016 | 62 (70.5%) |  | Stage I | 62 (70.5%) |
| 1975-1996 | 26 (29.5%) |  | Stage II | 1 (1.1%) |
| **Age at diagnosis (years)** |  |  | Stage IV | 13 (14.8%) |
| Median | 59.5 |  | Missing | 12 (13.6%) |
| Interquartile range (IQR) | 45.8-68.3 |  | **Tumor number** |  |
| **Sex** |  |  | Single | 71 (80.7%) |
| Female | 42 (47.7%) |  | Multiple | 17 (19.3%) |
| Male | 46 (52.3%) |  | **Surgical extent** |  |
| **Race** |  |  | GTR | 3 (3.4%) |
| White | 77 (87.5%) |  | PR | 27 (30.6%) |
| Black | 5 (5.7%) |  | No surgery | 51 (58.0%) |
| Other | 6 (6.8%) |  | Missing | 7 (8.0%) |
| **Marital status** |  |  | **Radiotherapy** |  |
| Married | 51 (58.0%) |  | Yes | 49 (55.7%) |
| Single | 23 (26.1%) |  | No/unknown | 39 (44.3%) |
| Widowed | 7 (8.0%) |  | **Chemotherapy** |  |
| Divorced | 2 (2.3%) |  | Yes | 50 (56.8%) |
| Separated | 1 (1.1%) |  | No/unknown | 38 (43.2%) |
| Missing | 4 (4.5%) |  |  |  |
| **Stage** |  |  |  |  |
| Localized | 46 (52.3%) |  |  |  |
| Reginal | 1 (1.1%) |  |  |  |
| Distant | 10 (11.4%) |  |  |  |
| Missing | 31 (35.2%) |  |  |  |
